# Study and Preparation of Multifunctional Poly(L-Lysine)@Hyaluronic Acid Nanopolyplexes for the Effective Delivery of Tumor Suppressive MiR-34a into Triple-Negative Breast Cancer Cells

**DOI:** 10.3390/ma13235309

**Published:** 2020-11-24

**Authors:** Jamila Djafari, Javier Fernández-Lodeiro, Hugo M. Santos, Julia Lorenzo, Sergi Rodriguez-Calado, Emilia Bértolo, José Luis Capelo-Martínez, Carlos Lodeiro

**Affiliations:** 1 BIOSCOPE Group, LAQV@REQUIMTE, Chemistry Department, NOVA School of Science and Technology, NOVA University Lisbon, Caparica Campus, 2829-516 Caparica, Portugal; j.djafari@fct.unl.pt (J.D.); j.lodeiro@fct.unl.pt (J.F.-L.); hmsantos@fct.unl.pt (H.M.S.); jlcm@fct.unl.pt (J.L.C.-M.); 2 PROTEOMASS Scientific Society, Rua dos Inventores, Madam Parque, Caparica Campus, 2829-516 Caparica, Portugal; 3 Institut de Biotecnologia i de Biomedicina, Universitat Autònoma de Barcelona, Campus Universitari, 08193 Bellaterra, Barcelona, Spain; sergi.rodriguez12@gmail.com; 4 Section of Natural and Applied Sciences, Canterbury Christ Church University, Canterbury CT1 1QU, UK; emilia.bertolo@canterbury.ac.uk

**Keywords:** polyplexes RNA, Poly-Lysine, triple negative breast cancer

## Abstract

Non-viral gene delivery using exogenous microRNAs is a potential strategy for fighting cancers with poor prognosis and which lack specific therapies, such as triple-negative breast cancer (TNBC). Herein we report the synthesis of six nontoxic electrostatic polymeric nanocapsules (P1 to P6) for microRNA delivery in TNBC cells. 1H Nuclear Magnetic Resonance (NMR) spectroscopy and Scanning Electron Microscopy (SEM) were used to characterize the nanopolyplexes, synthesized with Poly(L-Lysine) and hyaluronic acid (Ha). Studies on the activity of the ternary HA/PLI/miRNA-34 nanopolyplexes towards TNBC cell line MDA-MB-231 were conducted. The nanopolyplexes mediated intracellular restoration of tumor suppressor miR34a was evaluated by using Western blotting to quantify the expression level of the Bcl-2 protein. The results suggest that the P5, with a ratio PLI/Ha of 0.05, was the most promising for the delivery of miR-34a into TNBC cells; the P5 nanocapsules were able to reduce Bcl-2 expression at a protein level, and had an effect in the overall cell viability after 24 h treatment.

## 1. Introduction

Breast cancer is the second leading cause of cancer-related death after lung cancer, and is the most common diagnosed malignancy among women worldwide [1]. Significant progress has been made in diagnosing, monitoring and treating breast cancer. However, further research is needed to find ways to combat tumor relapses related to the onset of metastasis and resistance to conventional drugs; there is a need to develop new anticancer agents to promote the effectiveness of chemotherapy and reduce relapses [2,3].

Non-viral gene delivery using exogenous microRNAs (miRNAs) has emerged as a potential strategy for fighting against cancer. This kind of therapy has gained much attention, especially for more aggressive cancers which have worse prognosis and lack specific therapies, as is the case of the triple-negative breast cancer (TNBC). MiRNAs are conserved non-coding RNAs that negatively regulate gene expression by binding to the complementary sequence in the 3′-untranslated region (UTR) of target genes. MiR-34a has been involved in the inhibition of cell cycle progression and apoptosis of tumor cells; it also induces endothelial progenitor cell senescence and impedes angiogenesis [4]. In breast cancer, overexpression of miR-34a induces apoptosis of tumor cells, inhibits their proliferation and migration and reduces chemoresistance [5].

MiRNAs are short single-stranded sequences with unprotected 3′-hydroxy and 5′-phosphate ends, resulting in easy degradation by ribonucleases [6]; thus, they are only transiently expressed and have relatively short half-lives. Combined with a lack of tumor targeting, these are major limitations for their use in cancer therapy. Recently, nanocarriers have been suggested as a way to protect and target-deliver miRNAs at high levels directly to the tumor cells [7]. The ideal nanoparticle delivery vehicle requires the design of vectors sufficiently stable and nontoxic to transport the miRNA to the cytoplasm of the cancer cells. Many vectors have been developed based on the self-assembly of positively charged liposomes and polymers with the negatively charged nucleic acids, forming charged lipoplexes or polyplexes [8].

The small RNA miR-34a has been previously encapsulated in polyplexes formed by Poly-L-Lysine grafted with imidazole moiety and coated with PEGylated lipid vesicles [7]. The polyplexes, formed by self-assembly through electrostatic forces, yielded excellent results in terms of gene transport, non-degradation and intracellular delivery efficiency into gastric cancers cells [7]. The same research group also used this type of polyplexes to encapsulate Bcl-xL-specific shRNA-encoding plasmid DNA, targeting gastric cancer cells. Kim and co-workers designed and synthesized polyplexes formed by imidazole-poly-L-Lysine that were coated with hyaluronic acid to target gastric cancer cells through CD44, a glycoprotein over-expressed at their surface [2]. These electrically formed capsules can compact the nucleic acid and preserve it from the environment, increasing the delivery in the CD44- overexpressing tumor cells. The presence of imizadole groups allows the polyplexes to be sensitive to the pH of the medium. In fact, imidazole residues, which have a pka of 6, have a buffer capacity in the endosomal medium and can rapidly dissociate and release the nucleic acids in cancer cells.

Herein we present the synthesis of six new nontoxic electrostatic polymeric capsules for miR-34a delivery. The miR-34a-containing nanopolyplexes, formed by imidazole-poly-L-Lysine and hyaluronic acid, were developed to target cancer cells through the overexpressed CD44 receptor. The study of the impact of the nanopolyplexes for miR-34a delivery was also studied in a cellular model of triple-negative breast cancer.

## 2. Materials and Methods

Poly(L-Lysine hydrobromide) (PLL, Mw 10,000 Da) was purchased from Alamanda Polymers. N-(3-dimethylaminopropyl)-N-ethylcarbodiimide hydrochloride (EDC), 1-hydroxybenzotriazole hydrate (HOBt), 4-Imidazoleacetic acid, Sodium Hydroxide (NaOH), Hyaluronic acid sodium salt from Streptococcus equi (1.5–1.8 × 106 Da), 4-(2-Hydroxyethyl)piperazine-1-ethanesulfonic acid (HEPES) were purchased from Sigma Aldrich without further purification. Dialysis tubing benzoylated (cut off 2000) was purchased from Sigma Aldrich. Water was ultra-pure grade (type I).

MISSION^®^ microRNA Mimic (has-miR-34a-5p) was purchased from sigma Aldrich. The miR-34a sequence was: UGGCAGUGUCUUAGCUGGUUGU.

### 2.1. Synthesis of Imidazoled Poly-L-Lysine (PLI)

Poly-L-Lysine graft imidazole (PLI) was synthesized using N-(3-dimethylaminopropyl)-N-ethyl carbodiimide hydrochloride (EDC)/1-hydroxy benzotriazole hydrate (HOBt) coupling method from Poly(L-Lysine hydrobromide) (PLL)*,* by modification of a published method [2,8]. The molar ratio of carboxylic acid to ε-amine used was 0.50. 4-Imidazoleacetic acid (0.29 mmol) was dissolved in 10 mL of MilliQ water. Then, two molar excess of HOBt and EDC were added to the solution. The pH of the reaction was adjusted to pH 5 with 1N NaOH and let under stirring for 30 min. An aqueous solution of PLL (0.58 mmol of ε-amine, Mw 10,000, DPn 48) was added to the reaction, and the pH adjusted to pH 8.5. The reaction was left under stirring for 24 h at room temperature. Subsequently, the reaction was dialyzed against repeated MilliQ water changes using a dialysis membrane with a molecular weight cutoff of 2000, in order to remove the excess of reagents. The resulting solution was lyophilized and stored at −20 °C until use.

^1^H NMR PLI (400 MHz, D_2_O, ppm): δ = 7.80 − 7.29 (m, CH imidazole group), 4.18 (s, α–CH), 2.81 (m, ε–CH_2_), 1.82–1.05 (s, β–CH_2_, γ–CH_2_ and δ–CH_2_ in Poly-L-Lysine).

### 2.2. Preparation and Characterization of Ternary Polyplexes HA/PLI/MiRNA-34

Ternary polyplexes were prepared by electrostatic interaction modifying the N/P ratio in each experiment, following published methods [2,3,9]. N/P ratio corresponds to the moles of amine groups in each polymer (N) per moles of phosphate groups in miRNA-34a; N/P ration has an essential influence in the transfection efficiency [10]. PLI/miRNA-34a polyplexes were prepared in 10 mM HEPES buffer (pH 7.5). A fixed amount of miRNA-34a (3 µg) was mixed with PLI stock solution (1 mg/mL) at N/P ratios of 5, 10 and 20, and incubated at room temperature for 20 min. Then, a solution of hyaluronic acid (2 mg/mL) in 10 mM HEPES buffer (pH 7.5) was mixed with PLI/miRNA-3a polyplexes at 0.05 and 0.1 molar ratios of HA to PLI and incubated at room temperature for 20 min.

### 2.3. Scanning Electron Microscopy (SEM, Quanta650)

The SEM Quanta650 was used to examine the surface morphology of the synthesized polyplexes. For sample preparation, the polyplexes were diluted 1:10 in MilliQ H_2_O and mounted on a metal stub with an aluminum adhesive tape. After drying overnight, a sputtering device was used to coat the nanoparticles with a 5 nm width platinum layer under high vacuum. Samples were then analyzed with a 10 kV electron beam.

Gel retardation assays were conducted to evaluate the condensation ability of ternary HA/PLI/miRNA-34a polyplexes. The polyplexes contained 0.5 µg of miRNA-34a, and were prepared at N/P ratios of 5, 10 and 20. Electrophoresis was carried out with 1% agarose gel (*w*/*v*) containing ethidium bromide (0.05 µg/mL) in Tris-acetate-EDTA (TAE) buffer at 100 V for 30 min. The retardation of polyplexes was visualized under a UV lamp.

### 2.4. Cell Cytotoxicity Assay

Human triple-negative breast cancer cells MDA-MB-231 were cultured in RPMI media supplemented with 10% FBS (Gibco-BRL), and incubated at 37 °C in a humidified atmosphere with 5% CO_2_. Cells were seeded into a 96-well plate at a cell density of 3.0 × 103 cells per well and, then incubated for 24 h before the addition of the nanopolyplexes at 100 nM of encapsulated miRNA-34a. The growth inhibitory effect was measured after 24 h treatment employing the PrestoBlue assay [11]. PrestoBlue (10 μL; resazurin-based solution) was added to each well. After a two-hour incubation period (37 °C, 5 % CO_2_, 98 % humidity), the fluorescence was quantified using a fluorescent multilabel plate reader (Victor3, PerkinElmer) with excitation at λ = 531 nm and recorded at λ = 572 nm. Cell cytotoxicity was evaluated in terms of cell growth inhibition in treated cultures and expressed as a percentage of the control conditions. Each experiment was repeated at least three times, and each concentration was tested in at least three replicates.

### 2.5. Evaluation of the In Vitro Bcl-2 Suppression by MiR-34a Delivery

The nanopolyplexes mediated intracellular restoration of tumor suppressor miRNA-34a was evaluated by quantifying the expression level of Bcl-2 protein by Western blotting. MDA-MB-231 cells were seeded (2 × 105 cells per well) in 6-well plates and cultured for 24 h to reach 70% confluence at the time of transfection. Next day, the different polyplexes were added to each well at a final miRNA-34a concentration of 100 nM. After 72 h incubation, the medium was removed, cells were rinsed twice with 1 mL of phosphate-buffered saline (PBS, 10 mM, pH 7.4), and finally, cells were harvested and lysed with M-PER lysis buffer (Thermo Scientific, Waltham, MA, USA) following the manufacturer’s indications. After centrifugation at 12,000× *g* for 10 min, the supernatants were collected, and the concentrations of proteins were measured using Bradford’s reagent (Bio-Rad Laboratories, USA). The protein samples were denatured by boiling for 5 min and loaded onto SDS-PAGE gel for electrophoresis. The proteins were transferred onto PVDF (Polyvinilidene difluoride) membranes (Millipore, Darmstadt, Germany), and then incubated in blocking solution (5% non-fat dried milk) at room temperature for one hour. The anti-Bcl-2 (Cell Signalling Technology, Danvers, MA, USA) was added into blocking solution and incubated at 4 °C overnight. The membranes were subsequently incubated with the secondary goat anti-mouse antibody conjugated with HRP for 1 h. Protein expression was normalized against β-actin expression. Blotting images were acquired with the VersaDoc imaging system (Bio-Rad, Hercules, CA, USA) and analyzed by the software provided by the manufacturer.

## 3. Results and Discussion

**Figure 1:**
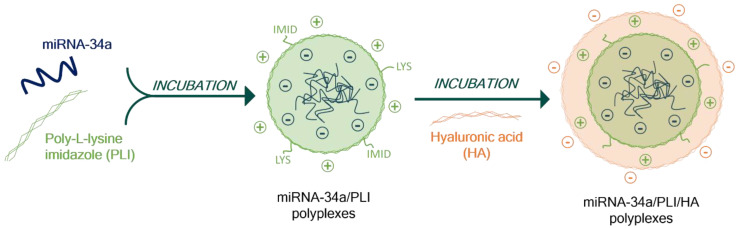
Schematic diagram of the key steps involved in the formation of the stable miRNA-34a/PLI/HA ternary polyplexes, showing the protonability at the endosomal environment, which allows intracellular gene delivery.

Figure 1 depicts the key steps involved in the formation of miRNA-34a containing polyplexes. The Imidazole Poly-L-Lysine (PLI) was synthesized by conjugation of carboxylic acid of 4-Imidazoleacetic acid with ε-amine of Poly-L-Lysine at a molar ratio of 0.5. We obtained a co-polymer composed of two different repetitive units, the lysine and the imidazole moiety, attached through amide linkages. ^1^H NMR analysis confirmed the successful conjugation of the imidazole group in the polymer by the presence of imidazole protons peaks at 7.80–7.29 ppm, similar to the literature.

**Figure 2:**
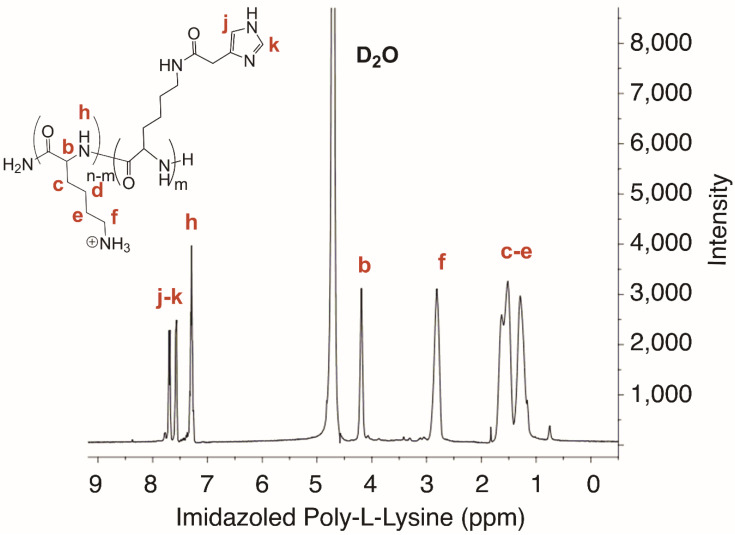
^1^H NMR spectrum of imidazole Poly-L-Lysine (PLI) in D_2_O at room temperature (400 MHz, D_2_O, ppm).

The percentage of imidazole moiety linked was calculated by NMR using characteristics imidazole proton α–CH peaks integration [12] (See Figure 2). We obtained a percentage of 46.9% of imidazole moiety for an input of 0.5%. The polymerization degree of the initial Poly-L-Lysine (PLI) is about 48 units with an average weight of 10,000 Da (g/mol). The copolymer obtained has an average composition of 22 imidazole units and 26 free lysines units, with an approximate molar mass of 8.5 kDa.

MiRNA-34a (3 µg) was encapsulated in PLI at different N/P ratio and covered with HA (see results, Section 2.2). Polyplexes were formed by electrostatic forces between the negative charges of RNA, positive charges of PLI and negative charge of HA. At endosomal pH (pH < 6), the nitrogen atoms of imidazole residues were protonated, and protons accumulate in the polyplexes leading to the release of miRNA-34a. All the polyplexes synthesized are summarised in Table 1.

**Figure 3:**
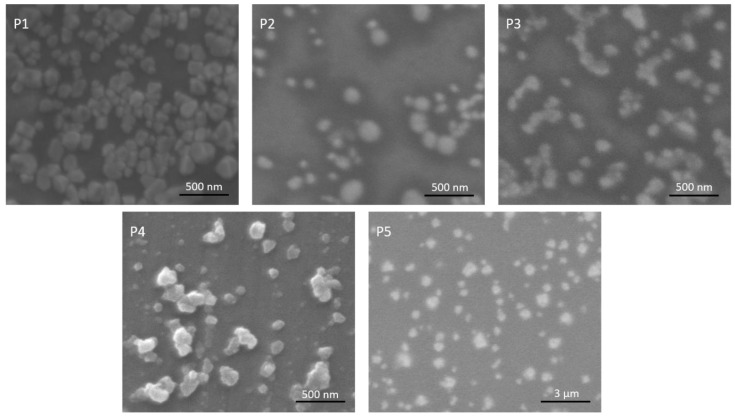
SEM images of the polyplexes at the different formulations. Average diameter and polydispersity index (PDI) was calculated from SEM images of the samples.

The surface morphology of the polyplexes was examined by scanning electron microscopy (SEM); results are shown in Figure 3. We can observe the spherical morphology of the nanoplexes is maintained in all the synthetic conditions. However, the different ratios used affect the size of the polyplexes. P5, with a higher N/P ratio (20) shows a substantial size increase compared with P1–P4 (see Table 2). Unfortunately, the high N/P and PLI/HA ratios of P6 made the sample not suitable for SEM preparation. The changes in size added to the variations in the composition of the polyplexes could affect their biological activity. P5 polyplex capsules, with an N/P ratio of 20 and PLI/HA ratio of 0.05, have an average diameter of 468.6 nm.

**Figure 4:**
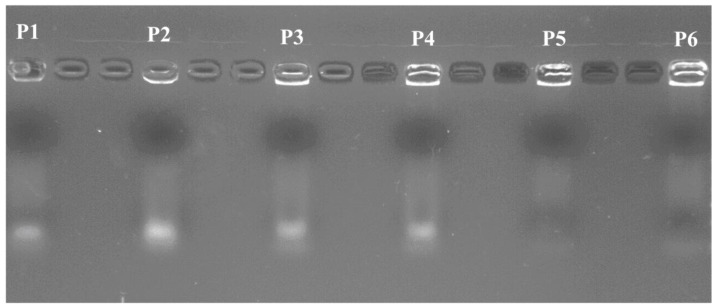
Gel retardation assay of the ternary polyplexes P1–P6, which possess different N/P ratios.

The N/P ratio of the polyplexes influences their condensation ability and transfection efficiency. The condensation ability of the ternary PLI/HA/miRNA-34a polyplexes was evaluated by gel retardation assay (see Figure 4). For P5 and P6, which have the highest quantities of PLI and HA of all the polyplexes studies (N/P ratio = 20), miRNA-34a was retarded, indicating that the miRNA-34a was completely condensed in PLA and HA. For polyplexes P1–P4, with N/P ratio of 5 (P1 and P2) and 10 (P3 and P4), the quantities of PLI and HA were not enough to condense fully miRNA-34a in the polyplexes.

**Figure 5:**
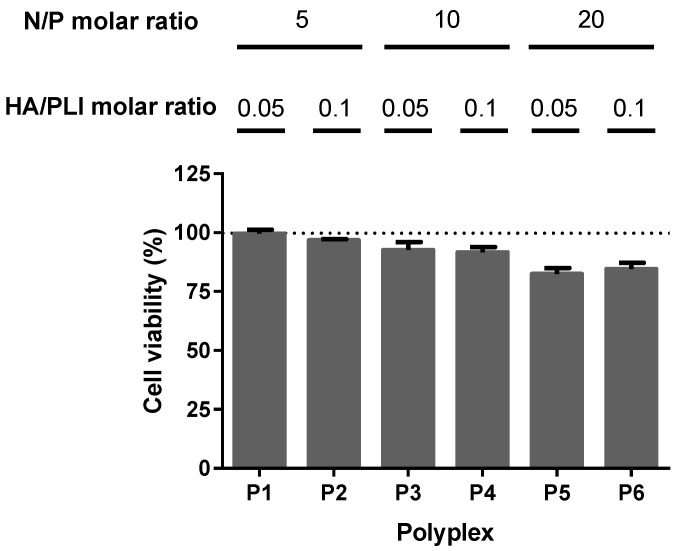
Effect on cell viability of miRNA-34a-loaded nanopolyplexes on triple negative breast cancer MDA-MB-231 cells. Cells were treated for 24 h with the indicated polyplex; N/P and HA/PLI molar ratios for each polyplex are included in the graph. The cell survival was determined by the PrestoBlue method as a percentage of the control. Data are expressed as the mean ± standard error of experiments performed in triplicate.

To investigate whether the difference in the N/P ratios and the HA concentration can effectively change the efficacy of miRNA-34a in silencing Bcl-2 expression, we first evaluated the cytotoxic effect of the different formulation of the nanopolyplexes in the triple negative breast cancer cell line MDA-MB-231; these cells were chosen because they overexpress the CD44 receptor. As shown in Figure 5, the lower N/P ratios (5 and 10) showed no inhibition of in vitro cell growth compared to the control group. The cells treated with a N/P ratio of 20 had little effect in cell inhibition growth, with cell viabilities around 80%. These results suggest that an increasing concentration of imidazole can protect the miRNA-34a delivery by buffering the endosomal medium and facilitating its delivery.

**Figure 6:**
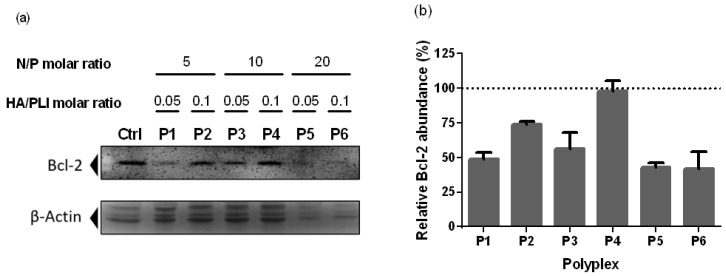
Protein expression levels of Bcl-2 in the MDA-MB-231 cells by Western blot analysis. Cells were treated for 24 h with the nanopolyplexes. (**a**) The Bcl-2 protein level was determined by Western blotting using anti-Bcl-2 antibody; N/P and HA/PLI molar ratios for each polyplex are included in the figure. (**b**) Graph showing the relative Bcl-2 abundance calculated as a percentage of the control. Each value represents the mean ± S.E.M. from duplicate determinations.

Bcl-2 has been identified as a potent regulator of apoptosis by preventing the permeability of the mitochondrial outer membrane [13]. An overexpression of Bcl-2 confers cancer cells’ resistance to chemotherapeutics agents such as doxorubicin, making it an exciting target for further cell sensibilization to current treatments [12]. Bcl-2 is targeted by miRNA-34a, and there is an epigenetic inactivation of miRNA-34a in cells derived from breast cancer [14,15]. In this work, the expression of Bcl-2 was evaluated at the protein level after the delivery of miRNA-34a in triple-negative breast cancer cells by the nanopolyplexes. Untreated cells were used as a negative control. Results are shown in Figure 6. Modest and non-downregulation of Bcl-2 expression was observed in the cells treated with P2 and P4. P2 and P4 have high PLI/HA ratio (0.1) but low to medium molecular N/P ratios (5 and 10 respectively). The results for P2 and P4 suggest that higher presence of HA in the coating may confer an excessive negative electrostatic charge to the complex, resulting in lower transfection efficiency of the nanopolyplexes and decreasing miRNA-34a uptake. Remarkable downregulation of Bcl-2 expression was noted in the cells treated with P1 and P3, both 0.05 PLI/HA ratio and 5 and 10 N/P molar ratios, respectively. The best result for downregulating Bcl-2 are from P5 and P6, which have the highest N/P ratio (20) and can completely condense the miRNA, suggesting that they are the best nanopolyplexes to deliver the miRNA-34a into the cells (Figure 6b).

The results suggest that HA helps in targeting the cells via CD44 recognition, but an equilibrium between the two polymers is needed for a correct internalization of the nanopolyplexes. Furthermore, the presence of a higher molar ratio of imidazole in the nanoparticles can overcome the deficiency of RNA delivery in the cells due to the complete condensation of the miRNA. P6 and especially P5 are promising nanopolyplexes for the delivery of miRNA-34a into triple-negative breast cancer cells; both nanopolyplexes were capable of reducing Bcl-2 expression at a protein level and had an effect in overall cell viability after 24 h treatment.

This is a very promising result that could make cancer cells more sensitive to chemotherapy or radiotherapy; this is of special relevance for TNBC patients, who tend to have a higher recurrence rate after diagnosis, a short disease-free interval, and reduced overall survival, especially due to the lack of targeted therapies [16]. The use of mi-RNA 34a in TNBC models has previously been reported as showing a reduction in the angiogenic process and in the proliferation, migration and onset of apoptosis or senescence. For effective miRNA replacement therapy, miRNAs must avoid early clearance and degradation in the bloodstream, travel through the extracellular matrix of the tumor, enter the target tumor cell and induce the silencing to enable gene regulation [17,18]. In this work, we loaded miR34a into PLI/HA nanopolyplexes to overcome these delivery challenges. Future studies should determine the potential therapeutic efficacy of nanopolyplexes P1, P6 and specially P5, in combination with traditional antitumoral drugs, using in vivo models.

## 4. Conclusions

We have successfully synthesized six new poly(L-lysine) polyplexes P1 to P6, containing encapsulated miR-34a. ^1^H NMR and SEM analyses confirm the formation of the polyplexes and the ratio of L-Lysine present. Cytotoxic studies of the six polyplexes, using with human triple-negative breast cancer cell line MDA-MB-231 yielded promising results, especially for polyplex P1 and P5, to a lesser extent, P6. P1 and P5 are promising nanopolyplexes for specific delivery of miRNA-34a into triple-negative breast cancer cells. The polyplex was able to reduce Bcl-2 expression at a protein level, showing an effect in the overall cell viability after 24 h treatment. These results suggest there is potential to use this non-toxic vector for selective miR-34a delivery in CD44 overexpressing tumors. Further studies with capsules P1, P5 and P6 are in progress.

## Figures and Tables

**Table 1:** MiRNA-34a/PLI/HA ternary polyplexes prepared with various N/P and PLI/HA ratios, in 10mM HEPES buffer. Each polyplex contained 3 µg of miRNA-34a.

Polyplexes	N/P Ratio	PLI/HA Ratio
P1	5	0.05
P2	5	0.1
P3	10	0.05
P4	10	0.1
P5	20	0.05
P6	20	0.1

**Table 2:** Polyplexes synthesised, average size (nm) and errors.

Polyplexes	Average Diameter (nm)	Standard Error of the Mean (SEM)
P1	121.46	6.02
P2	121.30	13.49
P3	125.41	5.30
P4	118.90	9.12
P5	448.07	36.68
